# MYC-targeted WDR4 promotes proliferation, metastasis, and sorafenib resistance by inducing CCNB1 translation in hepatocellular carcinoma

**DOI:** 10.1038/s41419-021-03973-5

**Published:** 2021-07-09

**Authors:** Peng Xia, Hao Zhang, Kequan Xu, Xiang Jiang, Meng Gao, Ganggang Wang, Yingyi Liu, Ye Yao, Xi Chen, Weijie Ma, Zhonglin Zhang, Yufeng Yuan

**Affiliations:** 1 Department of Hepatobiliary & Pancreatic Surgery, Zhongnan Hospital of Wuhan University, Wuhan, 430062 Hubei People’s Republic of China; 2 Clinical Medicine Research Center for Minimally Invasive Procedure of Hepatobiliary & Pancreatic Diseases of Hubei Province, Wuhan, 430062 Hubei People’s Republic of China

**Keywords:** RNA, Tumour biomarkers, Cell invasion

## Abstract

Hepatocellular carcinoma (HCC) is one of the most common malignancies worldwide. However, there still remains a lack of effective diagnostic and therapeutic targets for this disease. Increasing evidence demonstrates that RNA modifications play an important role in the progression of HCC, but the role of the N7-methylguanosine (m7G) methylation modification in HCC has not been properly evaluated. Thus, the goal of the present study was to investigate the function and mechanism of the m7G methyltransferase WD repeat domain 4 (WDR4) in HCC as well as its clinical relevance and potential value. We first verified the high expression of WDR4 in HCC and observed that upregulated WDR4 expression increased the m7G methylation level in HCC. WDR4 promoted HCC cell proliferation by inducing the G2/M cell cycle transition and inhibiting apoptosis in addition to enhancing metastasis and sorafenib resistance through epithelial-mesenchymal transition (EMT). Furthermore, we observed that c-MYC (MYC) can activate WDR4 transcription and that WDR4 promotes CCNB1 mRNA stability and translation to enhance HCC progression. Mechanistically, we determined that WDR4 enhances CCNB1 translation by promoting the binding of EIF2A to CCNB1 mRNA. Furthermore, CCNB1 was observed to promote PI3K and AKT phosphorylation in HCC and reduce P53 protein expression by promoting P53 ubiquitination. In summary, we elucidated the MYC/WDR4/CCNB1 signalling pathway and its impact on PI3K/AKT and P53. Furthermore, the result showed that the m7G methyltransferase WDR4 is a tumour promoter in the development and progression of HCC and may act as a candidate therapeutic target in HCC treatment.

## Introduction

Hepatocellular carcinoma (HCC) is one of the most common malignant tumours and the fourth leading cause of cancer deaths worldwide [1]. The occurrence and development of HCC is a complicated process involving a combination of aetiologies. Although targeted therapy has achieved breakthroughs in recent years, a large number of HCC patients still miss the best treatment opportunities due to late diagnosis [2, 3]. Thus, there is an urgent need to develop additional diagnostic markers and therapeutic targets for HCC, which requires further research to determine the occurrence and development of HCC [4–6].

Since discovering the first form of RNA modification, more than 150 different RNA modifications in various RNA molecules have been identified [7, 8], among which N7-methylguanosine (m7G) is the most abundant modification in the 5′ cap of mRNA [9–11]. With increased research in recent years, the m7G modification has been shown to have extensive effects on tRNA, rRNA, and mRNA and plays a vital role in various biological processes, including transcription elongation, pre-mRNA splicing, and mRNA translation [12–14]. However, the genetic characteristics and prognostic value of the m7G methyltransferase WDR4 in HCC remain unknown. Therefore, in the present study, we first used The Cancer Genome Atlas-Liver Hepatocellular Carcinoma (TCGA-LIHC) dataset and clinical data to assess WDR4 transcript levels in HCC and their impact on HCC patient prognosis. Mechanistically, we provided evidence that WDR4 is transcriptionally activated by c-MYC (MYC), facilitates cell proliferation and metastasis, and enhances sorafenib resistance by promoting CCNB1 translation in HCC. Thus, WDR4 is expected to be developed as a biomarker and potential therapeutic target for HCC patient prognosis.

## Results

### WDR4 is highly expressed in HCC and associated with poor HCC patient prognosis

**Fig. 1:**
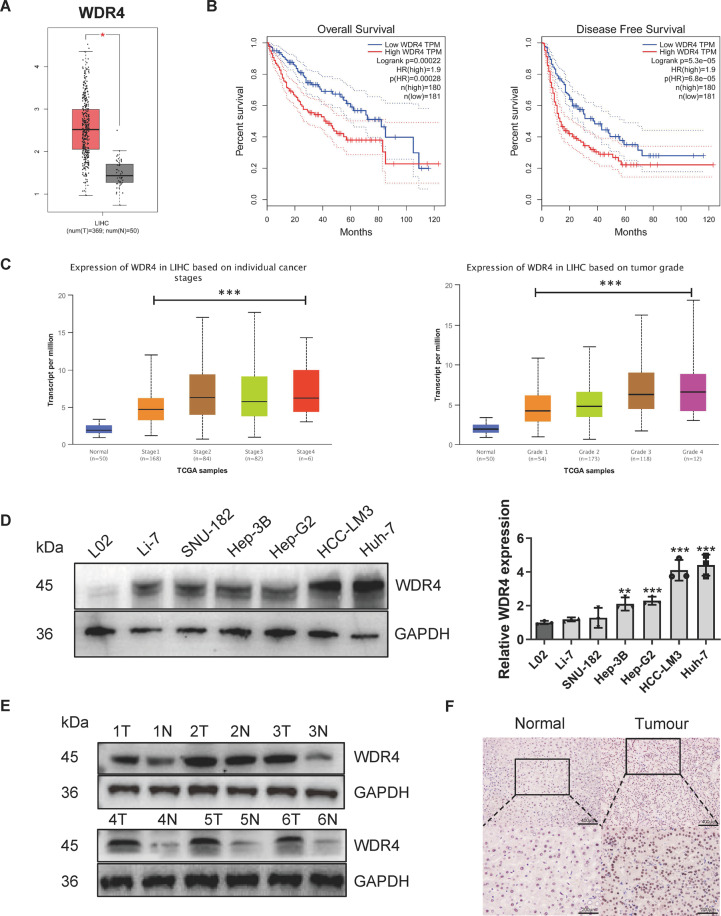
WDR4 expression is significantly increased in HCC. **A** Analysis of WDR4 transcript levels in HCC and normal tissues based on the TCGA database. **B** Overall and relapse-free survival curves of HCC patients. **C** WDR4 transcript levels are significantly related to HCC tumour stage and grade. **D** WDR4 expression in immortalized hepatocytes and six HCC cells. **E**, **F** Representative WDR4 expression and IHC results in 80 pairs of tumour tissues. *p < 0.05; **p < 0.01; ***p < 0.001.

Analysis of the TCGA-LIHC dataset showed that WDR4 transcript levels significantly increased in HCC (Fig. 1A). Kaplan−Meier survival analysis revealed that WDR4 overexpression was significantly related to the poor prognosis of HCC patients (Fig. 1B). UALCAN database analysis demonstrated that WDR4 transcript levels were significantly correlated with tumour grade and stage (Fig. 1C). To investigate the roles of WDR4 in HCC progression, we first evaluated the mRNA and protein levels of WDR4 in four HCC cell lines (Hep-3B, Hep-G2, HCC-LM3, and Huh-7) and observed that they were upregulated compared to those observed in hepatocytes (L02 cells; Fig. 1D). Additionally, the WDR4 mRNA and protein levels in tumours and adjacent normal tissues of 80 HCC patients were determined by qRT-PCR, Western blotting, and immunohistochemical (IHC) staining. WDR4 protein levels were significantly increased in HCC (Fig. 1E, F), while WDR4 mRNA levels were positively correlated with tumour size, TNM stage, BCLC stage, PVTT, and lymph node metastasis (Table S1). More importantly, univariate and multivariate analyses of clinicopathological characteristics revealed that high WDR4 expression in HCC correlated with shorter survival (Table S2). These results indicate that WDR4 is a promising candidate biomarker for the diagnosis and prognosis of HCC.

### WDR4 promotes HCC growth by inducing the G2/M cell cycle transition and inhibiting apoptosis

**Fig. 2:**
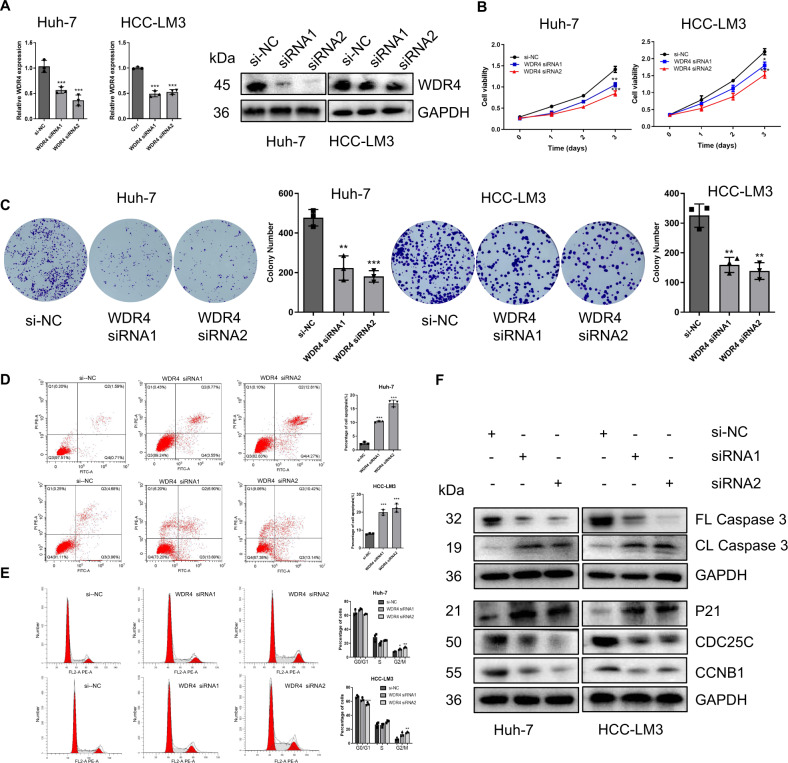
WDR4 promotes HCC cell growth. **A** Knockdown of WDR4 expression in Huh-7 and HCC-LM3 cells by siRNA. **B**, **C** CCK-8 and colony formation assays showing the proliferation ability of Huh-7 and HCC-LM3 cells. **D**, **E** Flow cytometry results showing the effect of WDR4 knockdown on apoptosis and cell cycle progression in Huh-7 and HCC-LM3 cells. **F** The expression of genes related to the cell cycle and apoptosis was detected by Western blotting. *p < 0.05; **p < 0.01; ***p < 0.001.

To further investigate the biological significance of WDR4 in HCC, we designed siRNA to knock-down WDR4 in Huh-7 and HCC-LM3 cells (Fig. 2A). The CCK-8 and clone formation assay results showed that WDR4 knockdown inhibited cell proliferation (Fig. 2B, C). Moreover, flow cytometry analysis showed that WDR4 knockdown caused cell cycle arrest in the G2/M phase and apoptosis (Fig. 2D, E). To further confirm the oncogenic effect of WDR4 in HCC, we constructed Li-7 cells overexpressing WDR4 (Fig. S1A). As expected, WDR4 overexpression promoted cell proliferation (Fig. S1B, S1C), induced the G2/M phase transition of the cell cycle, and inhibited cell apoptosis (Fig. S1D, S1E). Subsequently, we further assessed whether WDR4 regulates cell cycle- and apoptosis-related factors. Western blot analysis showed that WDR4 knockdown significantly upregulated P21 and downregulated CDC25C and CCNB1 (G2/M cyclin) protein levels. In addition, WDR4 knockdown significantly downregulated full-length (FL) caspase3 and upregulated cleaved-length (CL) caspase3 levels (Fig. 2F). These findings were supported by the results obtained through using WDR4-overexpressing cells (Fig. S1F) and indicated that WDR4 affects HCC cell growth by inducing the G2/M phase transition and inhibiting cell apoptosis.

### WDR4 promotes HCC cell migration and invasion by promoting EMT

**Fig. 3:**
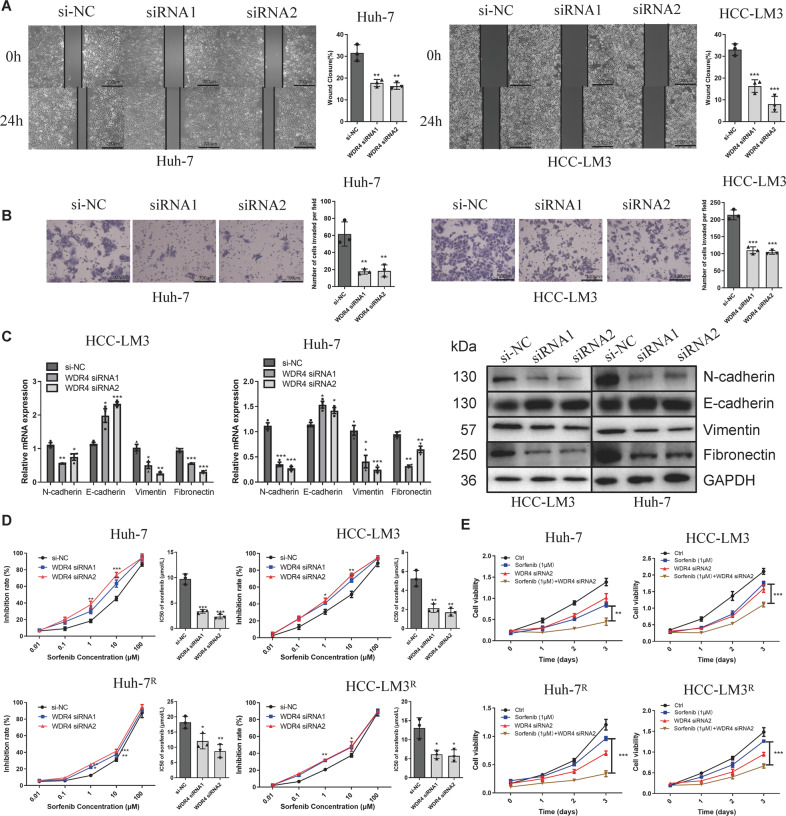
WDR4 promotes HCC invasion and migration through epithelial-mesenchymal transition (EMT). **A** After WDR4 knockdown, a scratch wound-healing motility assay was used to assess cell migration. **B** Transwell Matrigel invasion assay to measure the cell invasion ability. **C** qRT-PCR and Western blot results showed changes in EMT. **D** WDR4 knockdown in HCC-LM3, HCC-LM3^R^, Huh-7, and Huh-7^R^ cells increases their sensitivity to sorafenib. **E** CCK-8 assay results showing the proliferation ability of HCC-LM3, HCC-LM3^R^, Huh-7, and Huh-7^R^ cells. *p < 0.05; **p < 0.01; ***p < 0.001.

In subsequent experiments, transwell and scratch wound-healing assay results showed that WDR4 knockdown inhibited cell invasion and migration (Fig. 3A, B). Moreover, qRT-PCR and Western blot results demonstrated that WDR4 knockdown downregulated the expression of mesenchymal markers (N-cadherin, fibronectin, and vimentin) but upregulated that of E-cadherin (Fig. 3C). In addition, WDR4 overexpression significantly promoted cell invasion and migration (Fig. S1G, S1H, S1I). These results indicate that WDR4 can enhance the migration and invasion of HCC cells by promoting epithelial-mesenchymal transition (EMT). According to previous reports, EMT in tumours is a key process that mediates sorafenib resistance [15, 16]. Therefore, we next assessed the relationship between WDR4 and sorafenib resistance. WDR4 overexpression decreased the inhibition rate and increased the IC50 value of sorafenib in Li-7 cells, whereas inhibition rates were higher and the IC50 values were lower in HCC-LM3 and Huh-7 cells with siRNA-mediated WDR4 knockdown. Then, we constructed sorafenib-resistant Huh-7^R^, HCC-LM3^R^, and Li-7^R^ cells and observed that WDR4 overexpression significantly increased the IC50 value of sorafenib in Huh-7^R^ and HCC-LM3^R^ cells, while WDR4 knockdown significantly decreased the IC50 value in Li-7^R^ cells (Figs. 3D and S1J, S1K). Our results also showed that, in sorafenib-resistant cell lines, the expression of WDR4 was increased (Fig. S1L). Taken together, these results suggest that WDR4 promotes HCC resistance to sorafenib. Finally, we observed that the use of sorafenib and WDR4 inhibitors had a more pronounced inhibitory effect on HCC than the use of sorafenib alone in both sorafenib-resistant or normal HCC cells (Fig. 3E).

### WDR4 promotes tumour growth and metastasis in vivo

**Fig. 4:**
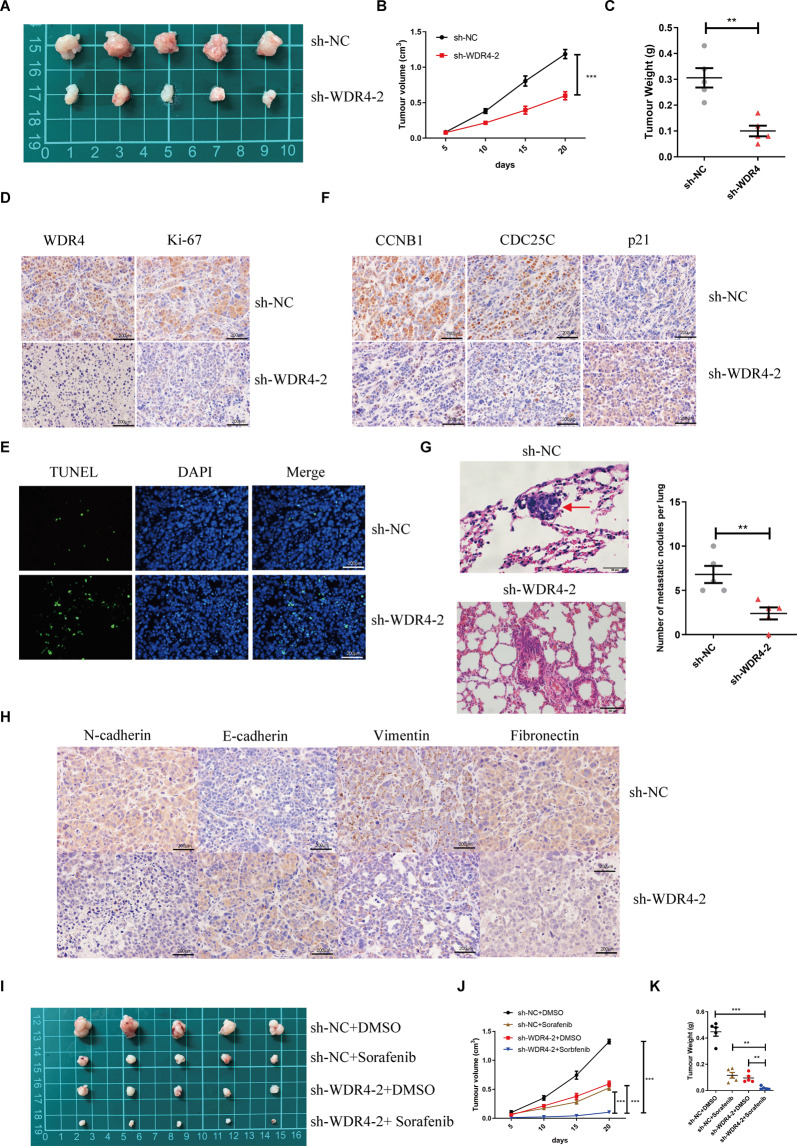
WDR4 promotes HCC cell growth and metastasis in nude mice. **A**, **B** Tumour growth curves of xenograft models established from control or stable WDR4-knockdown HCC-LM3 cells. **C** Assessment of the weight of the dissected tumours. **D** WDR4 and Ki-67 expression was determined by IHC analysis. **E** TUNEL expression levels were determined by IF analysis. **F** CCNB1, CDC25C, and P21 expression were determined by IHC analysis. **G** Metastasis in the lungs of nude mice injected with control or stable WDR4-knockdown HCC cells. **H** EMT-associated protein expression was determined by IF analysis. **I** Images of tumour xenografts of HCC-LM3 cells under different treatments. **J** Curves of tumour growth in each group. **K** Tumour weights were measured after collection. *p < 0.05; **p < 0.01; ***p < 0.001.

Subsequently, we established stable Huh-7 and HCC-LM3 cells transduced with lentiviruses harbouring an shRNA against WDR4 or a nonspecific scrambled shRNA (Fig. S2A). Compared to the sh-NC cells, sh-WDR4-2 cells exhibited decreased proliferation (Fig. S2B, S2C) and cell invasion ability (Fig. S2D, S2E), while cell apoptosis and G2/M cell cycle blockade were increased (Fig. S2F, S2G). To assess the effect of WDR4 on the growth and metastasis of HCC in vivo, we subcutaneously injected HCC-LM3 cells with stable WDR4 knockdown (sh-WDR4-2) or control cells (sh-NC) into nude mice. Tumour growth in the sh-WDR4-2 group was significantly slower than that observed in the sh-NC group (Fig. 4A, B), and the tumour weight was significantly reduced (Fig. 4C). In addition, the IHC and TUNEL staining results showed that WDR4 knockdown decreased the Ki67 proliferation index and increased the number of apoptotic cells. (Fig. 4D, E). The IHC results also showed that compared to sh-NC cells, CCNB1 and CDC25C expression in sh-WDR4-2 cells decreased, while that of P21 increased (Fig. 4F). To further substantiate the role of WDR4 in driving HCC metastasis, in vivo experiments with tail vein metastasis models were also conducted, with the results showing that WDR4 knockdown significantly reduced the formation of metastatic nodules in the lung (Fig. 4G). Moreover, after WDR4 knockdown, the expression of N-cadherin, vimentin, and fibronectin decreased, while that of E-cadherin increased (Fig. 4H). Next, we attempted to further confirm the role of WDR4 in sorafenib resistance in vivo and observed that either WDR4 knockdown or sorafenib treatment could inhibit tumour growth. However, the combination of WDR4 knockdown and sorafenib treatment resulted in the most significant inhibition of tumour growth (Fig. 4I, J, K). These results showed that WDR4 knockdown improves the antitumour effects of sorafenib.

### MYC activates WDR4 transcription

**Fig. 5:**
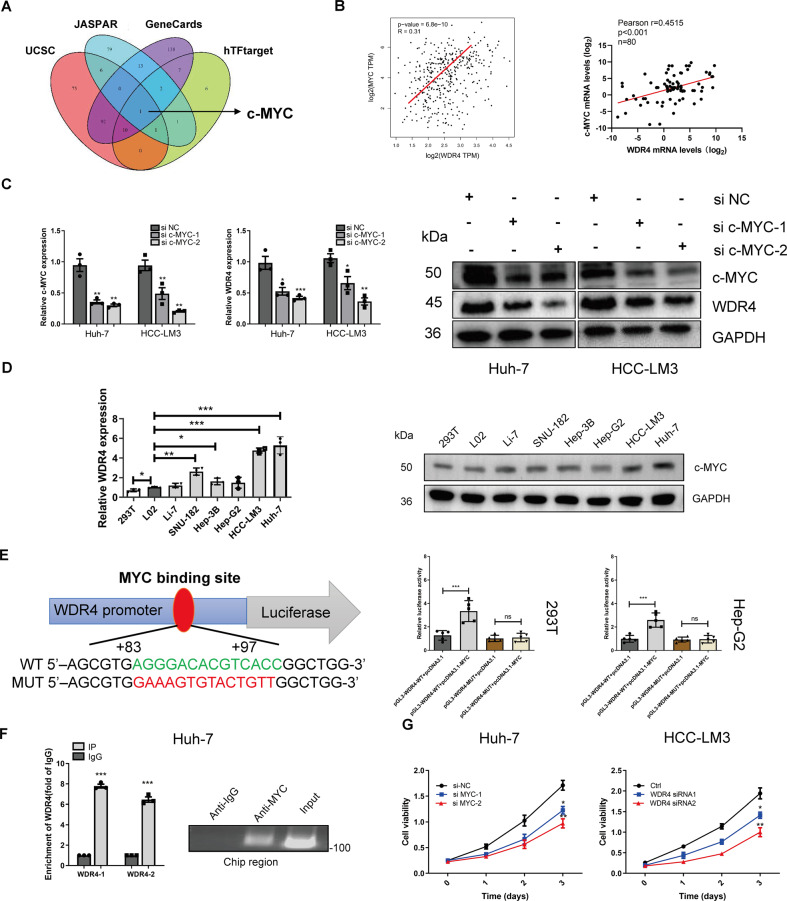
WDR4 is directly regulated by the transcription factor MYC. **A** Multidatabase joint analysis predicts MYC as a transcription factor of WDR4. **B** Pearson correlation analysis of WDR4 and MYC in the TCGA-LIHC dataset and HCC tissue samples. **C** After MYC knockdown, WDR4 mRNA and protein levels were significantly reduced. **D** MYC expression in different cell lines. **E** MYC binding sites in the wild-type (WT) and mutant (MUT) WDR4 promoters. Dual-luciferase reporter assays showing that MYC acts on the WDR4 promoter region. **F** ChIP results show that MYC binds to WDR4 (primers 1−2 are different primers for the WDR4 promoter). **G** CCK-8 assay results showing the proliferation ability of Huh-7 and HCC-LM3 cells. *p < 0.05; **p < 0.01; ***p < 0.001.

To identify the transcription factor that activates WDR4 transcription, we performed a combined analysis using the GeneCards, UCSC, hTFtarget, and JASPAR databases, the results of which identified only MYC as the key transcription factor regulating WDR4 expression (Fig. 5A and Table S6). The TCGA database correlation analysis results showed that WDR4 was positively correlated with MYC. Moreover, we measured MYC and WDR4 expression in 80 HCC tissue samples, and a subsequent scatter plot analysis showed a significantly positive correlation between the mRNA levels of WDR4 and MYC (Fig. 5B). We also observed that MYC knockdown reduced WDR4 expression in HCC cells (Fig. 5C). Additionally, MYC expression was higher in HCC cells than in normal liver and 293T cells (Fig. 5D). Therefore, we constructed 293T and HepG2 cell lines harbouring, a luciferase reporter vector containing WT or MUT MYC binding sequences. Dual-luciferase reporter assay results demonstrated that mutation of the MYC binding site abrogated the ability of MYC to promote WDR4 transcription (Fig. 5E). In addition, subsequent chromatin immunoprecipitation (ChIP) results confirmed that MYC binds to the WDR4 promoter region (Fig. 5F). Furthermore, we also observed that MYC knockdown in Huh-7 cells and HCC-LM3 cells, visibly decreased cell viability (Fig. 5G). In summary, our results revealed that the transcription factor MYC promotes WDR4 transcription by binding to the WDR4 promoter region.

### Identification of the WDR4 targets in HCC

**Fig. 6:**
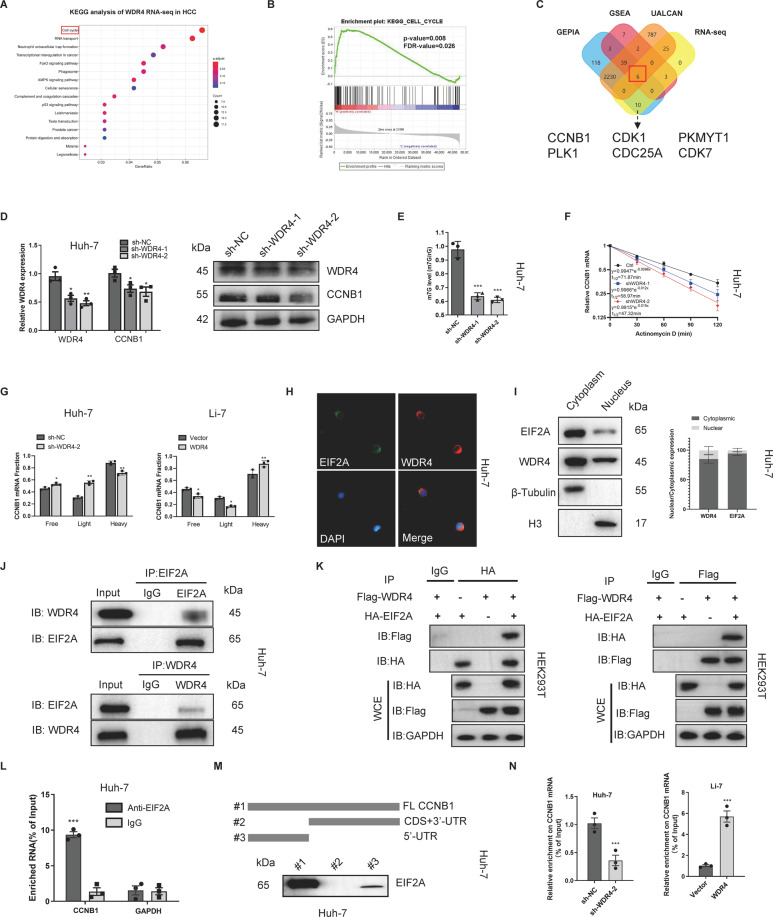
WDR4 is significantly associated with cell cycle signalling pathways. **A** KEGG analysis of RNA-seq data. **B** GEO data set (GSE105130) was used for GSEA. **C** Identification of WDR4-related genes through a multidatabase joint analysis. **D** Detection of CCNB1 expression in Huh-7 cells with stable WDR4 knockdown. **E** LC/MS results showing m7G methylation levels in Huh-7 cells. **F** Changes in CCNB1 mRNA stability after actinomycin D treatment of Huh-7 cells. **G** Relative distribution of CCNB1 mRNA across the polysome fractions in HCC cells. **H** Representative immunofluorescence images showing WDR4 and EIF2A colocalization. **I** Western blot analysis of WDR4 and EIF2A using nucleocytoplasmic separation in Huh-7 cell lines. **J**, **K** Endogenous co-IP assay and exogenous co-IP assay results showing the binding of WDR4 and EIF2A. **L** RIP-qPCR results showing the correlation between EIF2A and CCNB1 mRNA levels. **M** Schematic representation of CCNB1 mRNA depicting fragments used for biotin pulldown assays. **N** RIP-qPCR results showing the binding of EIF2A and CCNB1 mRNA levels in the presence and absence of WDR4. *p < 0.05; **p < 0.01; ***p < 0.001.

To elucidate the potential molecular mechanism by which WDR4 affects the growth and metastasis of HCC, we performed RNA sequencing (RNA-seq) in Huh-7 and HCC-LM3 cells transduced with sh-NC or sh-WDR4-2. Volcano plots were generated to identify differentially expressed genes in HCC-LM3 and Huh-7 cells (Fig. S3A), and a Venn diagram was constructed to show the intersection of differentially expressed genes between these cell types (Fig. S3B). KEGG enrichment analysis of the RNA-seq data revealed WDR4 to be significantly related to the cell cycle pathway (Fig. 6A), as did the gene ontology (GO) analysis results, which also showed it to be associated with the structural constituents of ribosomes and methyltransferase activity (Fig. S3C, S3D). Subsequently, we performed gene set enrichment analysis (GESA) to analyze genes with coexpressed WDR4 in GEO data (GSE105130), the results of which also indicated enrichment of the cell cycle pathway (Fig. 6B). To further support the results of our study, we used strict cut-off values (*R* > 0.3, *P* < 0.05) to screen genes coexpressed with WDR4 in the GEPIA and UALCAN databases and used the genes in the intersection for further analysis. Through joint analysis, we identified CCNB1, CDK1, PKMYT1, PLK1, CDC25A and CDK7 as WDR4-related genes in the cell cycle pathway (Fig. 6C), with a heatmap and correlation analysis showing the correlation between genes (Fig. S3E, S3F). Then, we observed that stable WDR4 knockdown induced a decrease in CCNB1 mRNA and protein levels, while no changes were observed after CCNB1 knockdown (Figs. 6D and S4A). In addition, WDR4 knockdown did not cause significant changes in other WDR4-related genes (results not shown). In summary, our results identified CCNB1 as a downstream target of WDR4 for further research.

### WDR4 promotes CCNB1 mRNA translation and stability

To determine whether WDR4 regulates CCNB1 through m7G-dependent methylation, we used LC-MS/MS and dot blot analysis to assess the m7G methylation level of Huh-7 cells. After WDR4 knockdown, the overall level of m7G methylation in HCC cells was significantly reduced (Figs. 6E and S4B, S4C), and the m7G site on CCNB1 mRNA could not be detected by Me-RIP analysis (Fig. S4D). Thus, WDR4 promotes m7G but does not regulate CCNB1 mRNA in an m7G-dependent manner in HCC. As WDR4 has been reported to be the primary tRNA m7G methyltransferase and regulates the mRNA translation process, we speculated that WDR4 regulates the translation of CCNB1 [10]. However, interestingly, we observed that the stability of CCNB1 mRNA was reduced after WDR4 knockdown (Fig. 6F). The polysome profiling results showed that WDR4 knockdown resulted in an increased association of CCNB1 mRNA with free and light ribosomal fractions and a decreased association with heavy ribosomal fractions. Furthermore, these findings were supported by the results obtained through using WDR4 overexpressing cells (Fig. 6G). These findings indicate that WDR4 promotes CCNB1 mRNA translation and stability.

### WDR4 enhances CCNB1 translation by promoting EIF2A binding to CCNB1 mRNA

We next sought to understand the mechanism through which WDR4 promotes CCNB1 translation, hypothesizing that WDR4 may interact with translation initiation factors to promote CCNB1 expression. Therefore, we performed IP and LC-MS/MS analyses to identify the proteins bound by WDR4. EIF2A plays a central role in maintaining what is generally considered a rate-limiting step in mRNA translation [17]. Interestingly, the silver staining and MS analysis results showed that CCNB1 interacts with EIF2A (Fig. S4E). In addition, IF and nucleocytoplasmic separation results showed that WDR4 and EIF2A are primarily co-located in the cytoplasm, where translation takes place (Fig. 6H, I). Their binding was confirmed by endogenous co-IP and exogenous co-IP experiments (Fig. 6J, K). Subsequently, we verified the binding of EIF2A to CCNB1 mRNA by RIP-qPCR (Fig. 6L). Furthermore, we constructed a truncated 5′UTR and CDS+3′-UTR fragments of CCNB1 mRNA and performed RNA pulldown assays, the results of which showed that the 5′UTR RNA fragment bound EIF2A, while the CDS+3′-UTR fragment exhibited reduced signal intensity (Fig. 6M). These data indicate that EIF2A directly binds to the CCNB1 5′UTR. Subsequently, we conducted RIP-qPCR, and the results indicated that WDR4 can increase the interaction between CCNB1 mRNA and EIF2A protein (Fig. 6N). Finally, our results showed that after silencing EIF2A, WDR4 knockdown could not reduce the stability of CCNB1 mRNA, and the positive effect on CCNB1 mRNA translation was lost (Fig. S4F, S4G, S4H). In summary, our results indicate that the effect of WDR4 on CCNB1 mRNA translation is EIF2A-dependent and that WDR4 enhances CCNB1 translation by promoting EIF2A binding to CCNB1 mRNA.

### Ectopic CCNB1 expression ameliorates the tumour-suppressive effect of WDR4 deficiency in HCC cells

**Fig. 7:**
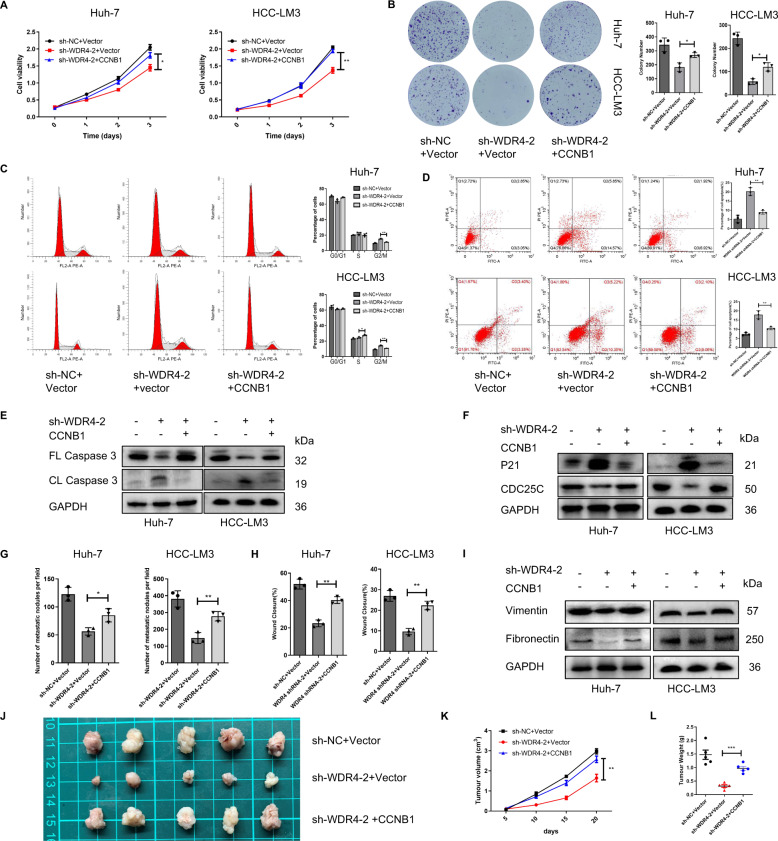
WDR4 promotes HCC cell growth and metastasis through CCNB1. **A**, **B** CCK-8 and colony formation assays showing the proliferation ability of Huh-7 and HCC-LM3 cells. **C**, **D** Flow cytometry results showing the effect of CCNB1 on apoptosis and cell cycle progression. **E**, **F** The expression of genes related to the cell cycle and apoptosis was detected by Western blotting. **G** Transwell Matrigel invasion assay of cell invasion ability. **H** Scratch wound-healing motility assay of cell migration. **I**. The expression of genes related to EMT was detected by Western blotting. **J** Assessment of the size and weight of the dissected tumours. **K** Curves of tumour growth in each group. **L** Tumour weights were measured after collection. *p < 0.05; **p < 0.01; ***p < 0.001.

Our results showed that CCNB1 overexpression rescued the effects of WDR4 knockdown on HCC cell growth (Figs. 7A, B and S5A), cell cycle progression, apoptosis (Fig. 7C, D), cell invasion, and migration (Figs. 7G, H and S5B, S5C). In addition, we observed that CCNB1 overexpression could reduce the effects of WDR4 on apoptosis markers (caspase3) and cell cycle markers (P21 and CDC25) (Fig. 7E, F). Moreover, CCNB1 overexpression restored the expression of some EMT markers (vimentin and fibronectin) as well as sorafenib resistance (Figs. 7I and S5D). These results were also verified in vivo (Fig. 7J, K, L). Taken together, these findings further confirmed that WDR4-mediated HCC cell proliferation and metastasis functions at least in part via CCNB1 expression.

### CCNB1 promotes PI3K/AKT phosphorylation and P53 ubiquitination in HCC

**Fig. 8:**
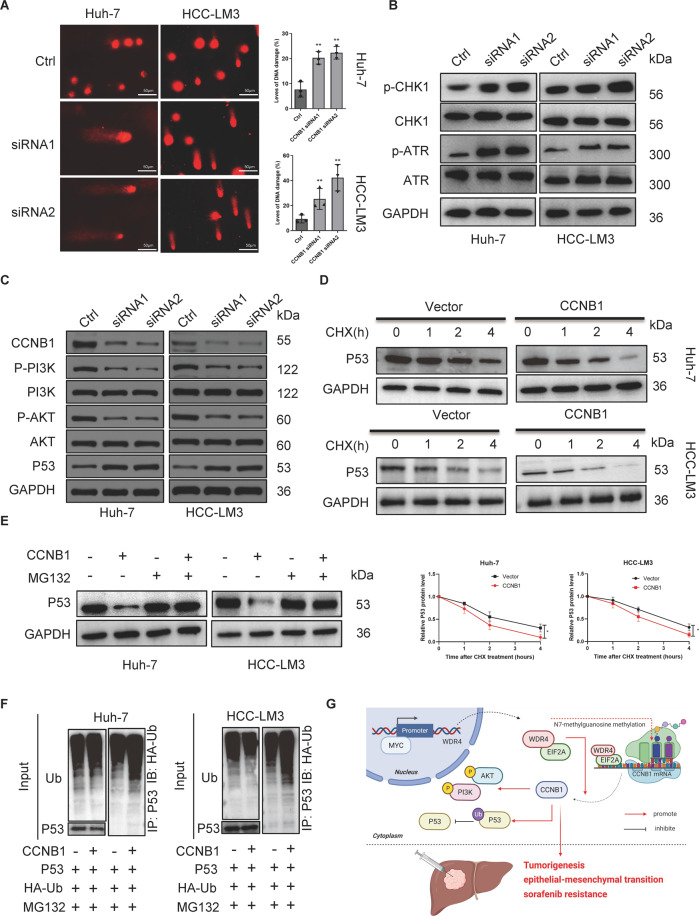
CCNB1 promotes PI3K/AKT phosphorylation and P53 ubiquitination. **A** DNA damage levels in Huh-7 cells and HCC-LM3 cells. **B**, **C** Western blot assay of cells transfected with control siRNA or CCNB1 siRNA. **D**, **E** Western blot analysis of P53 expression. **F** Ubiquitination assays of cells transfected with the CCNB1 expression plasmid. **G** Model of the regulatory mechanisms of WDR4 in HCC. *p < 0.05; **p < 0.01; ***p < 0.001.

TCGA database analysis showed that CCNB1 was highly expressed in HCC and strongly correlated with the prognosis of HCC patients (Fig. S6A, S6B). Furthermore, analysis of the HPA database showed that CCNB1 expression in HCC tissues was significantly higher than that observed in normal liver tissues (Fig. S6C). CCNB1 overexpression has been reported to aggravate the growth and metastasis of HCC cells and is associated with the AKT/PI3K and P53 signalling pathways [18–21]. Therefore, we assessed the function of CCNB1 in HCC and its correlation with PI3K, AKT, and P53. For the first time, our results indicate that CCNB1 knockdown promotes DNA damage in HCC (Fig. 8A) and promotes phosphorylation of the DNA damage marker proteins CHK1 and ATR (Fig. 8B). Moreover, our results showed that CCNB1 knockdown inhibited phosphorylation of the proteins PI3K and AKT (Fig. 8C). It has been published that signalling through AKT-PI3K is essential for EMT [22]. We added relevant experiments to verify the effect of CCNB1 on EMT. The results showed that vimentin and fibronectin were significantly downregulated after CCNB1 knockdown, but there was no significant effect on E-cadherin and N-cadherin (Fig. S6D). Additionally, we observed that after treatment with a PI3K-AKT pathway inhibitor (PI3K-IN-1, MCE), knockdown of CCNB1 could not downregulate vimentin and fibronectin (Fig. S6E). These results indicate that CCNB1 promotes EMT through the PI3K-AKT pathway.

We also tested the effect of CCNB1 on P53 stability using cycloheximide (Fig. 8D). The proteasome inhibitor MG132 antagonized the decrease in P53 protein levels caused by CCNB1, indicating that CCNB1 downregulates P53 expression by promoting its proteasome degradation (Fig. 8E). In addition, we observed that CCNB1 can induce P53 protein degradation by promoting the ubiquitination of P53 (Fig. 8F). Finally, the IHC staining results showed that HCC patients with higher WDR4 expression showed higher CCNB1 expression, while those with higher CCNB1 expression showed lower P53 expression (Fig. S6F).

## Discussion

As an emerging hotspot in RNA modification research, m7G methylation has been shown to be involved in multiple cellular processes and is closely related to the dysregulation of mRNA, tRNA, and rRNA [13, 23, 24]. The RNA methyltransferase WDR4 plays a key role in translation and tumour progression [10, 25]. In the present study, for the first time, we investigated the biological functions and molecular mechanisms underlying WDR4 activity in HCC. We observed that WDR4 is highly expressed in HCC and significantly associated with the prognosis of HCC patients, indicating that WDR4 may play an important role in the development of HCC as a proto-oncogene. Clinical data showed that WDR4 is associated with tumour metastasis, and our results further showed that the transfer of WDR4 is primarily mediated by EMT and that high WDR4 expression can cause sorafenib resistance. Our findings showed the efficacy of WDR4 inhibitors on tumour growth in HCC patients and showed the possibility of their combined use with sorafenib, paving the way to develop new treatment strategies and therapeutic drugs for HCC in the future.

MYC is one of the most common oncogenic transcriptional regulators and affects almost all cellular processes, with MYC expression being elevated or dysregulated in up to 70% of human cancers [26]. One of the primary biological functions of MYC is to promote cell cycle progression. The evidence to date suggests that MYC triggers selective gene expression amplification to promote cell growth and proliferation [27]. Therefore, abnormally amplified MYC often leads to the overexpression of several carcinogenic molecules, which accelerates tumour progression. Through multidatabase joint analysis, we determined that MYC promotes HCC progression at least in part by promoting WDR4 transcription. These findings provide new biological insights into the MYC signalling pathway.

According to previous reports, WDR4 has a wide range of effects on mRNA translation, especially those related to the cell cycle [10]. These observations are consistent with our findings, which showed for the first time that WDR4 enhances the translation of CCNB1 mRNA by promoting its binding to EIF2A. However, our research also has some limitations. WDR4 mainly affects the m7G methylation of tRNA, and the reduction of m7G methylation modification level in human tRNA may lead to the degradation of one or more specific tRNA types, resulting in reduced or abnormal translation [28, 29]. tRNA deregulation induces protein synthesis errors, which have been correlated with accelerated tumour growth kinetics. The regulation of CCNB1 mRNA stability and translation level by WDR4 may also be achieved by specific tRNA m7G methylation, but this needs to be clarified by more sophisticated instruments.

CCNB1 is a key molecule that regulates G2/M phase progression and can affect tumour growth and metastasis. A large number of proteins are phosphorylated by the CCNB1-CDK1 complex before entering mitosis. It has been reported that reduction in CCNB1 levels leads to polyploidy in DNA damage-induced senescence [30]. In the present study, for the first time, we identified CCNB1 as a downstream target of WDR4 in HCC and demonstrated that CCNB1 reduces DNA damage and promotes PI3K/AKT phosphorylation and P53 ubiquitination. PI3K/AKT functions play vital roles in quiescence, survival, and growth in normal physiological circumstances, as well as in various pathological disorders, including cancer. Possible mechanisms by which the PI3K/AKT axis contributes to oncogenic transformation include stimulation of proliferation, EMT reprogramming, and survival, as well as suppression of autophagy and senescence [16, 22]. Our findings confirm that CCNB1 inhibitors can inhibit PI3K/AKT phosphorylation, which may have a great impact on EMT. Furthermore, our results showed that WDR4 regulates EMT in HCC in part through CCNB1.

The tumour suppressor P53 plays a central role in tumour suppression, and the precise regulation of P53 function is essential for determining cell fate. Among the multilayered mechanisms that control P53 function, posttranslational modifications represent an effective and precise method, including phosphorylation, ubiquitination, acetylation, and methylation [31, 32]. Our results showed that CCNB1 affects P53 stability by promoting P53 ubiquitination. In addition, ubibrowser results showed that P53 has multiple ubiquitination sites. We speculate that CCNB1 promotes P53 protein ubiquitination through the E3 ligase CCNB1IP1 (Table S7), although further investigation is needed to test this possibility.

In summary, in the present study, we elucidated the MYC/WDR4/CCNB1 signalling pathway and its impact on PI3K/AKT and P53. These findings provide new ideas for the study of m7G methyltransferases. RNA methyltransferases may have more complex functions, which poses a new challenge to the study of RNA methylation.

## Materials and methods

### Bioinformatics analysis

In our research, we downloaded HCC patient data from the TCGA-LIHC data set and a GEO data set (GSE105130). Gene Set Enrichment Analysis (GSEA) v3.0 software was used for gene enrichment analysis, and FunRich was used for Gene Ontology (GO) analysis. All online analysis website URLs are shown in Table S3.

### Cell culture

Huh-7, Li-7, HCC-LM3, SUN-182, Hep-3B, and Hep-G2 cell lines were purchased from the Chinese Academy of Sciences Cell Bank (Shanghai, China). L02 cells were purchased from Fuxiang Biotechnology Company (Shanghai, China). L02, Huh-7, HCC-LM3, and Hep-G2 cell lines were cultured in DMEM medium plus 10% fetal bovine serum (FBS). Hep-3B cell lines were cultured in MEM medium plus 10% FBS, 1% non-essential amino acids, and 1% sodium pyruvate. Li-7 cell lines were cultured in MEM medium plus 10% FBS. SUN-182 cell lines were cultured in RPMI-1640 medium plus 10% FBS. All cell lines maintained in a 5% CO2 incubator at 37 °C.

### Tissue samples

Eighty pairs of HCC tissues and non-tumour liver tissues were collected from HCC patients at Zhongnan Hospital of Wuhan University (Hubei, China) from April 2014 to March 2019, and all patients provided written informed consent. In this study, none of the patients had preoperative chemotherapy or radiation therapy. Two pathologists confirmed the HCC tissues. The study was performed according to the Declaration of Helsinki and was approved by the Ethics Committee of Zhongnan Hospital of Wuhan University (KELUN2020100).

### RNA extraction and real-time quantitative PCR (qRT-PCR)

TRIzol (Takara, Dalian, China) was used to extract total RNA, and an enzyme kit was used for reverse transcription. qRT-PCR was performed using 2× ChamQ Universal SYBR qPCR Master Mix (Vazyme). Table S4 lists the sequences of all PCR primers used.

### Western blotting

RIPA buffer (Solarbio, Beijing, China) was used to extract total protein. After electrophoresis and blocking with milk, the PVDF membrane was incubated with the appropriate antibody. The antibodies used are shown in Table S5. An ECL chemiluminescence imaging system (Tanon-5200, Shanghai, China) was used to detect ECL signals.

### Immunohistochemistry (IHC) analyses

For IHC analysis, the paraffin sections were placed in an oven at 65 °C for 2 h and then deparaffinized to water. Then the slices were microwaved in EDTA buffer for repair and placed in a 3% hydrogen peroxide solution incubated for 10 min. After blocking with 5% BSA for 20 min, about 50 μl diluted primary antibody was added to each slice at 4 °C overnight. Add 100 μl horseradish peroxidase (HRP)-conjugated secondary antibody of the related species to each slice for 50 min. Add 100 μl diaminobenzidine (DAB) solution to each slice, and then stained with hematoxylin before dehydration in a graded alcohol series and xylene. The IHC-stained tissue sections were scored by two pathologists who were blinded to the clinical parameters.

### Immunofluorescence (IF) analyses

For IF analysis, cells were washed three times with phosphate-buffered saline (PBS) and fixed with 4% paraformaldehyde for 20 min. HCC cells were then permeabilized with 0.5% NP-40 in PBS for 20 min and blocked with 5% bovine serum albumin for a duration of 30 min. Next, cells were incubated with primary antibodies for 2 h and incubated with secondary antibodies for 1 h. For the nuclei, cells were stained with DAPI (ASPEN, AS1075). Finally, confocal microscopy (OLYMPUS, IX51) was used for image acquisition.

### Plasmids, siRNA transfection, and shRNA

The coding sequence of WDR4 was cloned into the vector pcDNA 3.1, which was transfected into cells with Lipo3000 (Thermo Fisher, NY, USA). siRNA was designed and synthesized by Qingke Biological and delivered into HCC cells with GenMute^TM^ siRNA Transfection Reagent (SignaGen, US). Gene Create Co. (Wuhan, China) packaged the negative control and shRNA into lentiviruses. Then, the transduced cells were treated with 2 µg/ml puromycin to generate stable WDR4 knockdown cell lines. Table S4 lists the sequences of siRNA and shRNA used in the present study.

### Nude mouse tumour xenograft model and lung metastasis models

Five-week-old BALB/c nude mice were ordered from Wuhan University Experimental Animal Centre (Wuhan, China). All mice were bred and monitored under specific pathogen-free (SPF) conditions. Control or HCC-LM3 cells with stable knockdown of WDR4 were subcutaneously injected into the left axillary or tail vein of BALB/c nude mice. Then, the mice were divided into groups, randomly using a table of random numbers (five mice per group). The sample size is estimated to detect a significant difference among the groups. During the experiments, the investigator was blinded for the sample group allocation. The mice were sacrificed 20 days after cell injection, the tumours were collected, their weights were measured, and the mouse lungs were removed for haematoxylin & eosin (HE) staining. The animal study was approved by the Ethics Committee of Zhongnan Hospital.

### Cell proliferation assays

Cell proliferation was detected by using the CCK-8 assay and the colony formation assay as previously described [33].

### Scratch wound-healing motility assay

Cell migration was determined using a scratch wound-healing motility assay as previously described [33].

### Transwell invasion assay

Cell invasion was determined using a Transwell invasion assay as previously described [33].

### Cell apoptosis analysis

HCC cells were grown to 80% confluence on six-well plates, after which serum-containing medium was replaced by medium without serum. After 24 h of cell incubation without serum, all cells were collected. Cell apoptosis was detected using Annexin V-FITC apoptosis detection kit (Lianke, Hangzhou, China). Briefly, HCC cells were stained with Annexin-V-FITC/Propidium Iodide (PI) followed by Cytoflex flow cytometer (Beckman, China) analysis within 5 min of staining. Data were analyzed using FlowJo software (version 10.6.0, Ashland, OR, USA).

### Cell cycle analysis

For cell cycle synchronization, HCC cells were grown in 0.05% serum for 48 h to synchronize in the G0 phase and then washed and release into the medium with 10% FBS to release into the cell cycle synchronously. Cell cycle was detected by Cytoflex flow cytometer (Beckman, China), about the manufacture’s protocol of the Cell Cycle Staining Kit (Lianke, Hangzhou, China).

### Generation of sorafenib-resistant cells

Sorafenib-resistant cell lines, Huh-7^R^, HCC-LM3^R^, Li-7^R^ were induced by continuous treatment of Huh-7, HCC-LM3, Li-7 cell lines with sorafenib up to 10 μmol/L to imitate acquired resistance. Viable cells remaining attached to the dish at the end of the third round of drug treatment were considered sorafenib-resistant and collected for experiments. Sorafenib was purchased from MCE (Shanghai, Chian).

### Reporter vector construction and luciferase reporter assay

Dual‐luciferase reporter assay was used to detect the interaction between MYC and the WDR4 promoter region. WDR4 wild-type (WT) and WDR4 mutant (MUT, with mutated binding sites) sequences were synthesized and inserted into the pGL3-basic vector. Lipofectamine 3000 was used for transfection, and 48 h after transfection, the Dual Luciferase® Reporter Assay System (Promega) was used to measure luciferase activity.

### Chromatin immunoprecipitation (ChIP)

ChIP was performed to explore the potential binding between the promoter region of WDR4 and MYC in HCC cells. The cells were fixed with formaldehyde, and the lysate was sonicated. The ultrasonicated samples were divided into the IgG group (mixed with 10 µL IgG antibody) and IP group (mixed with 10 µL WDR4 antibody) and subjected to immunoprecipitation. After elution and decrosslinking, qRT-PCR analysis was performed. All primer sequences are shown in Table S2.

### RNA-sequencing (RNA-seq)

Briefly, RNA integrity was assessed using the Agilent 2100. Sequencing libraries were generated using VAHTS mRNA-seq v2 Library Prep Kit for Illumina. According to the manufacturer’s instructions, the libraries were sequenced on an Illumina NovaSeq platform. Differentially expressed genes were defined as *P*-value < 0.05 and absolute log2 fold change > 1.

### Dot blot assay

Dot blot assay was used to clarify the differences in m7G methylation in different samples. The RNA sample was spotted on a nylon membrane and UV cross-linked, and 10 mL of PBST containing 5% BSA was added to seal the membrane. Subsequently, the m7G antibody was used for incubation, and the ECL system (Tanon, USA) was used to detect the signal. Finally, methylene blue staining was used as an RNA loading control.

### LC-MS/MS quantification of m7G

LC-MS/MS assay was used to clarify the differences in m7G methylation in different samples. Adding buffer, S1 nuclease, Alkaline Phosphatase, and Phosphodiesterase I into 1 μg RNA, the mixture was incubated at 37 °C. After the RNA was digested into nucleosides completely, the mixture was extracted with chloroform. The resulting aqueous layer was collected for analysis with LC-ESI-MS/MS (UPLC, ExionLC AD; MS, Applied Biosystems 6500 Triple Quadrupole).

### Methylated RNA immunoprecipitation (Me-RIP) assay

Me-RIP assay was used to investigate the m7G site on CCNB1 mRNA in Huh-7 cells. Fragmentation buffer was added to the RNA sample to fragment the RNA into approximately 100-nucleotide fragments, which were then purified with an RNase MiniElute Kit. A/G magnetic beads (MCE) and m7G antibody were used for specific enrichment and antibody incubation. After elution, reverse transcription, and amplification, qPCR was performed on the RNA input, m7G-IP fraction, and IgG-IP fraction, and the corresponding CT value was calculated.

### Polysome profiling

Polysome profiling was used to detect mRNA translation. In short, after treating the cells with CHX for 3 min, lysis buffer was added for lysis. Subsequently, the lysate was added to the prepared 10−50% sucrose density gradient. The extract was fractionated at 35 000 rpm for 3 h, and then the fraction was recovered using a gradient fractionator. RNA was extracted from each part, and the translation status of CCNB1 mRNA on the polysome fraction was determined by qRT-PCR.

### Actinomycin D chase assay

Actinomycin D (Act D) chase assay was performed for mRNA stability as previously described [34].

### Co-immunoprecipitation (co-IP) and LC-MS/MS analysis

The co-IP assay was performed as described previously [33]. The protein samples were analyzed using Q Exactive Plus mass spectrometer (Thermo Scientific, Massachusetts, USA) coupled with the UltiMate 3000 RSLC nanosystem (Thermo Fisher).

### Subcellular fractionation assay

The separation of nucleus−cytoplasm in Huh-7 cell lines was performed following the manufacturer’s instructions of the PARIS Kit (Invitrogen). β-Tublin and H3 were employed as the indicators for quantification of the fractions.

### RNA immunoprecipitation (RIP) assay

RIP assay was utilized to investigate the relationship between EIF2A and CCNB1 in Huh-7 cells. According to the manufacturer’s instructions, RIP assays were carried out using the Magna RIP RNA binding protein immunoprecipitation kit (Millipore, MA, USA). In brief, HCC cells were fully lysed by a lysis buffer containing a mixture of RNase inhibitors and protease inhibitor cocktail. After centrifugation at 4 °C, 12 000 rpm for 15 min, transfer the mixture to a new EP tube centrifuge at 3000 rpm for 1 min to clean the magnetic beads twice. The mixture was mixed with magnetic beads and 5 μL of antibody overnight at 4 °C. After purification, RNA was evaluated by qRT-PCR analysis.

### RNA pulldown assay

RNA pulldown assay was also used to investigate the interaction between the EIF2A protein and CCNB1 each fragment in Huh-7 cells. In brief, the plasmids carrying full-length or different CCNB1 mRNA deletion mutants, were first linearized and transcribed in vitro. The biotinylated RNA was incubated with streptavidin magnetic beads (Invitrogen) overnight at 4 °C according to the manufacturer’s instructions (Thermo Scientific). RNase inhibitor and cell lysates were incorporated into each binding reaction on ice for 1 h. The RNA−protein mixture was washed briefly with sodium dodecyl sulfate buffer three times. Finally, The RNA−protein mixture was identified by western blot using an anti-EIF2A antibody.

### Alkaline comet assay

DNA damage was determined by an alkaline Comet assay. Briefly, alkaline comet assay was detected by the manufacturer’s protocol of the Reagent Kit for Single Cell Gel Electrophoresis Assay (ELK Biotechnology, Wuhan, China). First of all, lysis cells with pre-chilled lysis buffer. After lysis, gels were transferred to an electrophoresis chamber filled with alkaline unwinding buffer (1 mmol/L EDTA, 300 mmol/L NaOH) for 20–60 min at room temperature. Electrophoresis was conducted with the same buffer in 25 V for 30 min. After adding buffer solution (0.4 mmol/LTris-HCl) for neutralization, add PI dye solution and EB dye solution to stain for 10 min in the dark. The results of the comet experiment were analyzed using ImageJ-Opencomet software. Briefly, 100 cells were randomly selected in each sample for measurement, and the percentage of tail DNA content to head DNA content (TailDNA%) was calculated.

### Ubiquitination assay

Ubiquitination assay was performed as described previously [34].

### Statistical analysis

All statistical analyses were performed with SPSS 21.0 (IBM SPSS Statistics for Windows, Version 21.0. Armonk, NY, USA: IBM Corp) and Prism 7.0 (GraphPad Software, La Jolla, CA, USA). The student’s t-test was used for comparisons between two groups, and survival analysis was performed using Kaplan−Meier analysis. Multiway analysis of variance was performed to assess data obtained from the CCK-8 assays and tumour growth experiments, and Spearman’s rank correlation analysis was used to evaluate the correlation between genes. All experiments were repeated three times, and *P* < 0.05 was considered to indicate a significant difference.

## Supplementary information

Supplementary figures S1−S6

Supplementary tables S1−S5

Supplementary table S6

Supplementary table S7

## Data Availability

RNA-seq data have been deposited in GEO under accession number GSE169394. The data that support the findings of this study are available from the corresponding author upon reasonable request.

## Code availability

The R packages “pheatmap” and “corrplot” were used to generate heat maps and correlation heat maps. The R package “VennDiagram” was used to generate Venn diagram. The R package “edgeR” was used for differential gene expression analysis. No custom code was used for analysis.
