# Supplementary material for: MYC-targeted WDR4 promotes proliferation, metastasis, and sorafenib resistance by inducing CCNB1 translation in hepatocellular carcinoma
Source: Cell Death Dis. 2021 Jul 9;12(7):691. doi: 10.1038/s41419-021-03973-5 (PMC8270967; doi:10.1038/s41419-021-03973-5)
Supplement: Supplementary file 1 — Supplementary figures S1−S6 [file 41419_2021_3973_MOESM1_ESM.pdf]

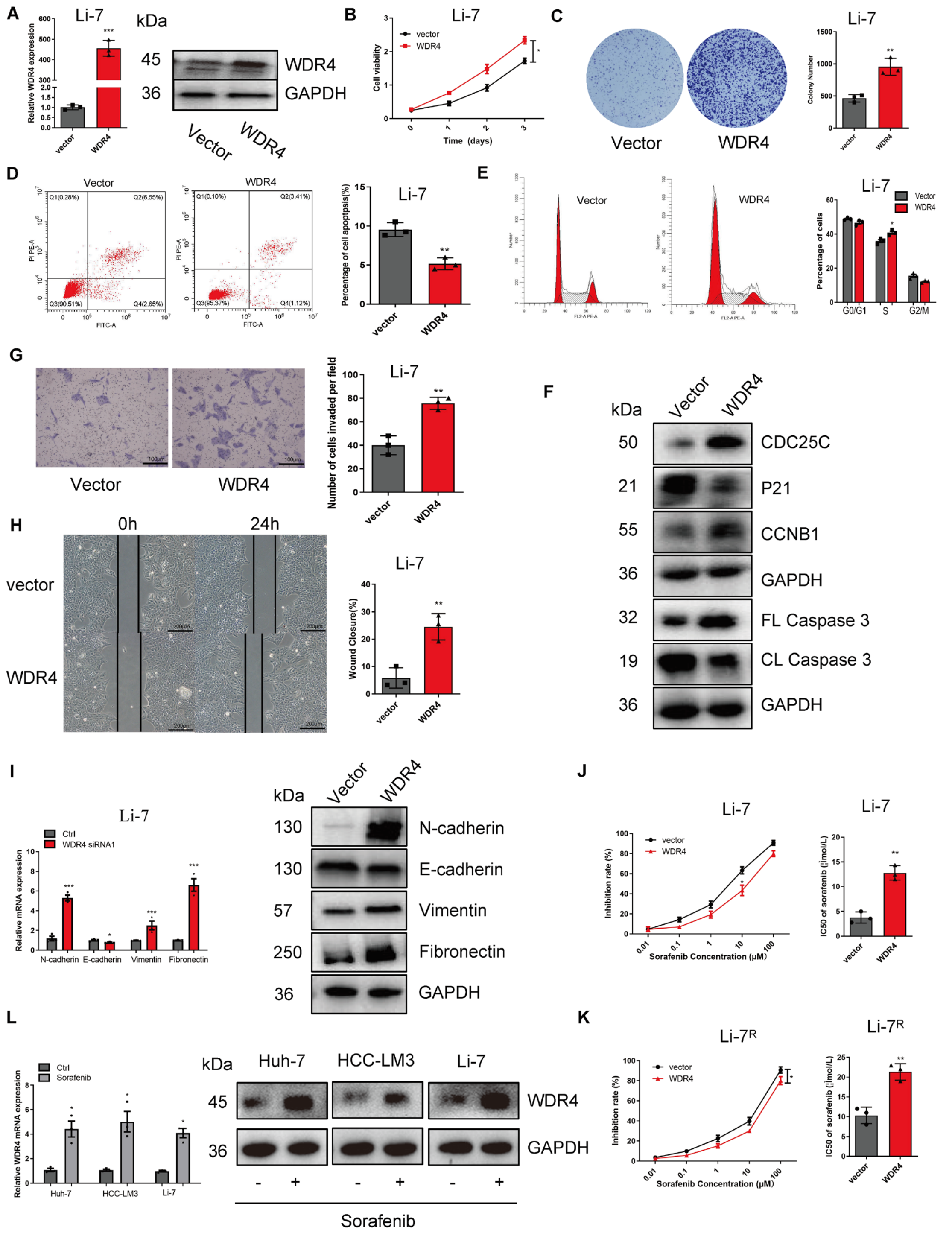

Figure S1. WDR4 overexpression increases HCC cell growth and metastasis

A The WDR4-overexpressing plasmid was transformed into the Li-7 cell line. B, C CCK-8 and clone formation experiments showed that WDR4 functions in cell proliferation. D, E Flow cytometry results showed changes in apoptosis and cell cycle progression. F The expression of proteins related to the cell cycle and apoptosis was detected by Western blotting. G, H The effect of WDR4 overexpression on cell invasion and migration. I, J, K The effect of WDR4 overexpression on EMT pathway proteins and sorafenib resistance. L Human HCC cell lines were treated with sorafenib, and the protein expression of WDR4 was assayed by qRT-PCR and western blot.

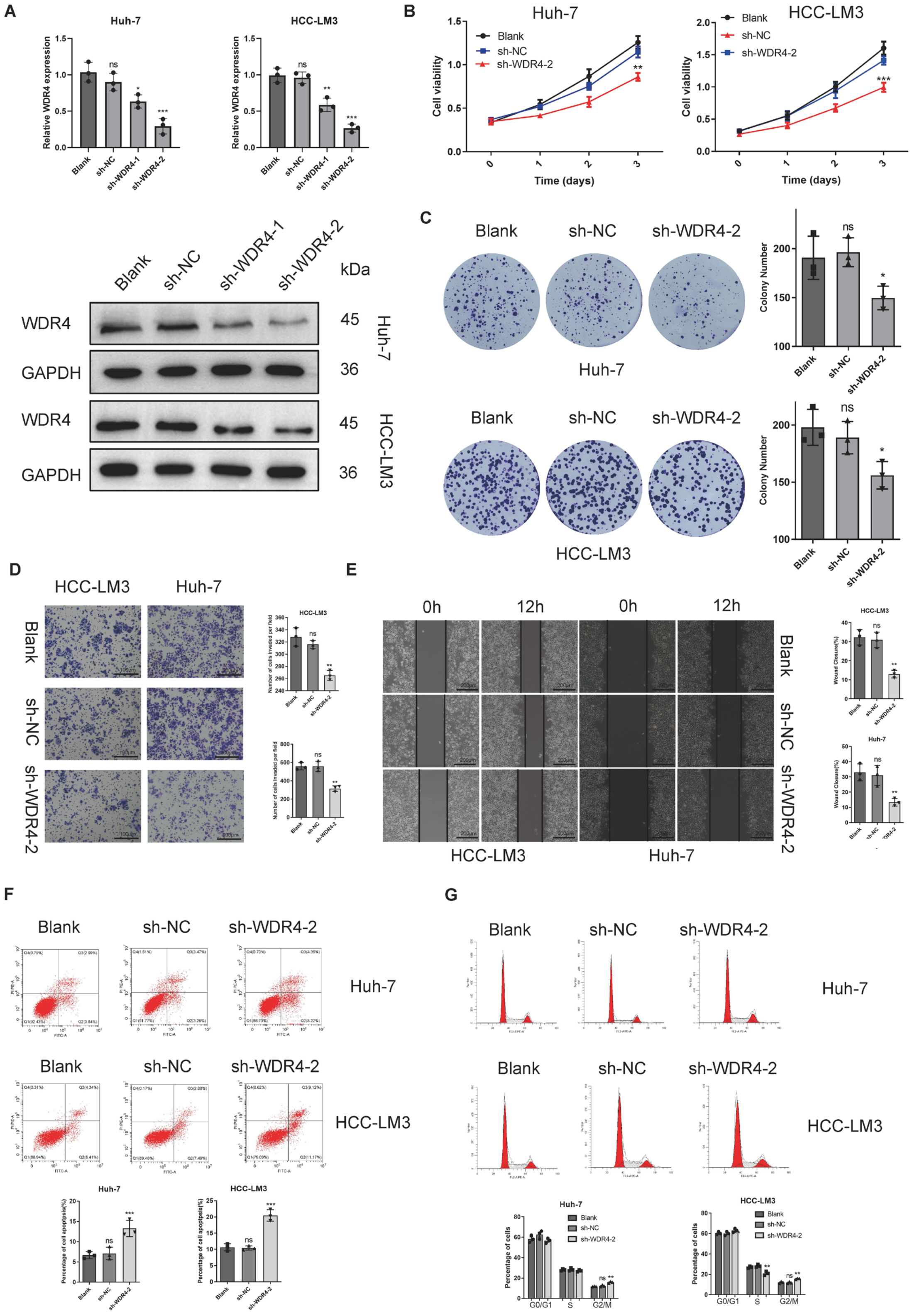

Figure S2. Effect of WDR4 shRNA transduction on the phenotype of HCC cells

A Knockdown of WDR4 expression in Huh-7 and HCC-LM3 cells by shRNA. B, C CCK-8 and colony formation assays showing the proliferation ability of Huh-7 and HCC-LM3 cells. D Transwell Matrigel invasion assay to measure cell invasion ability. E Scratch wound-healing motility assay was used to determine cell migration. F, G Flow cytometry results showing the effect of WDR4 knockdown on apoptosis and cell cycle progression in HCC cells.

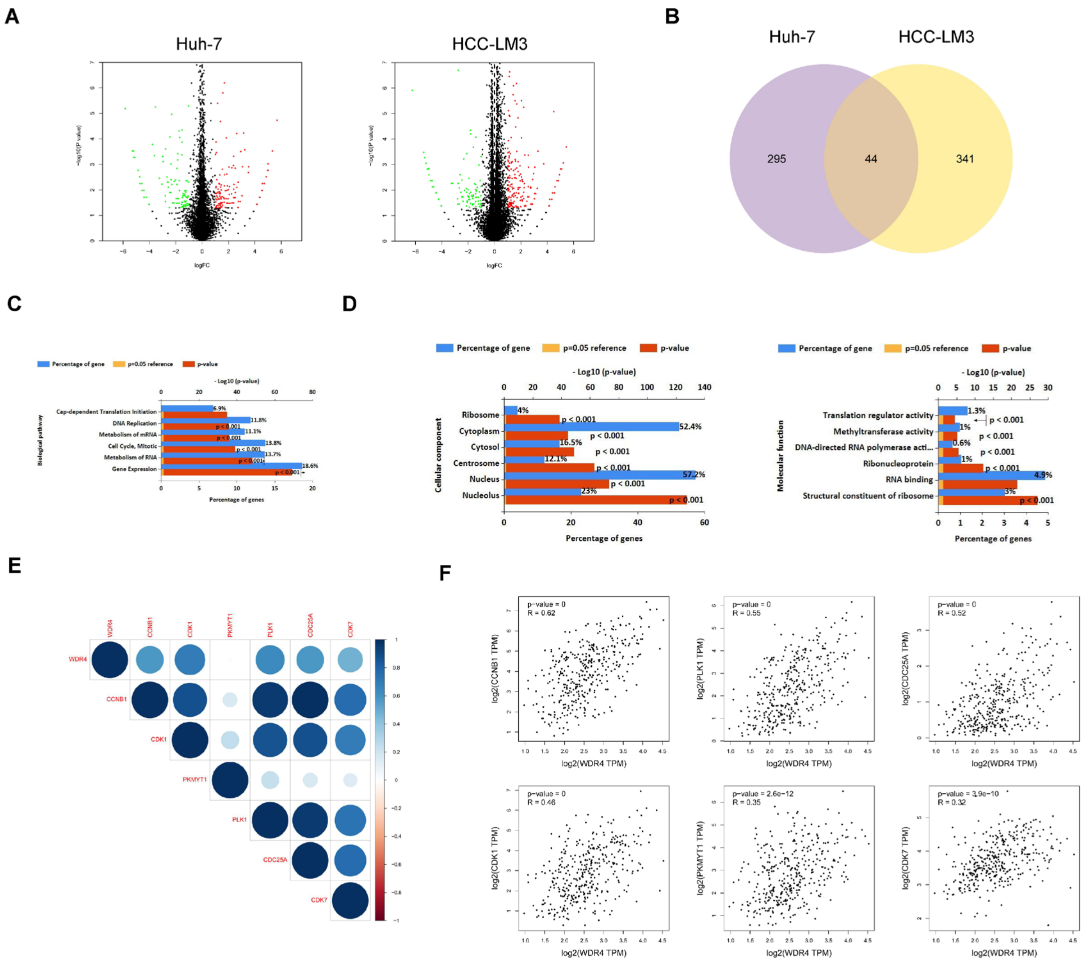

Figure S3. Correlation between WDR4 and related genes

A Volcano plots showing differentially expressed genes in HCC-LM3 and Huh-7 cells. B A Venn diagram showing the intersection of differentially expressed genes between HCC-LM3 and Huh-7 cells. C, D GO analysis showing that WDR4 is related to cell cycle progression, ribosomes and methyltransferase activity. E, F Correlation heat map and correlation analysis showing the correlation between the relevant genes enriched in the cell cycle pathway and WDR4.

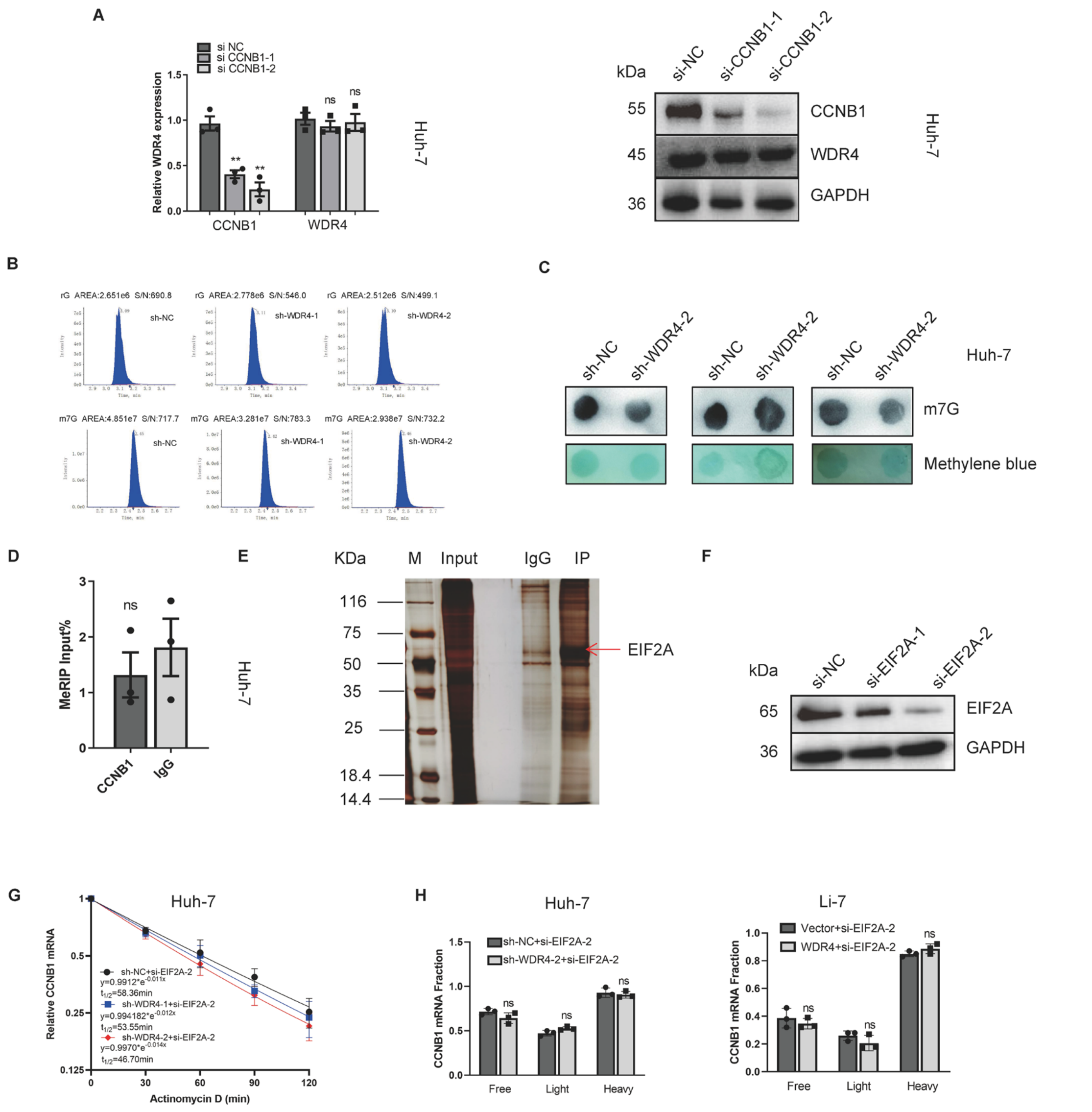

Figure S4. WDR4 enhances CCNB1 mRNA translation by recruiting EIF2A

A Detection of WDR4 expression in the Huh-7 cell line with CCNB1 knockdown. B LC/MS results showing the m7G methylation level. C Dot blot showing changes in m7G methylation. D Me-RIP results showing the m7G level of CCNB1 mRNA. E WDR4-interacting proteins are shown by silver staining. F Detection of EIF2A expression in the Huh-7 cell line. G Changes in CCNB1 mRNA stability after actinomycin D treatment. H Polysome profiles of control and WDR4 knockdown cells and polysome profiles of vector- and WDR4-overexpressing cells.

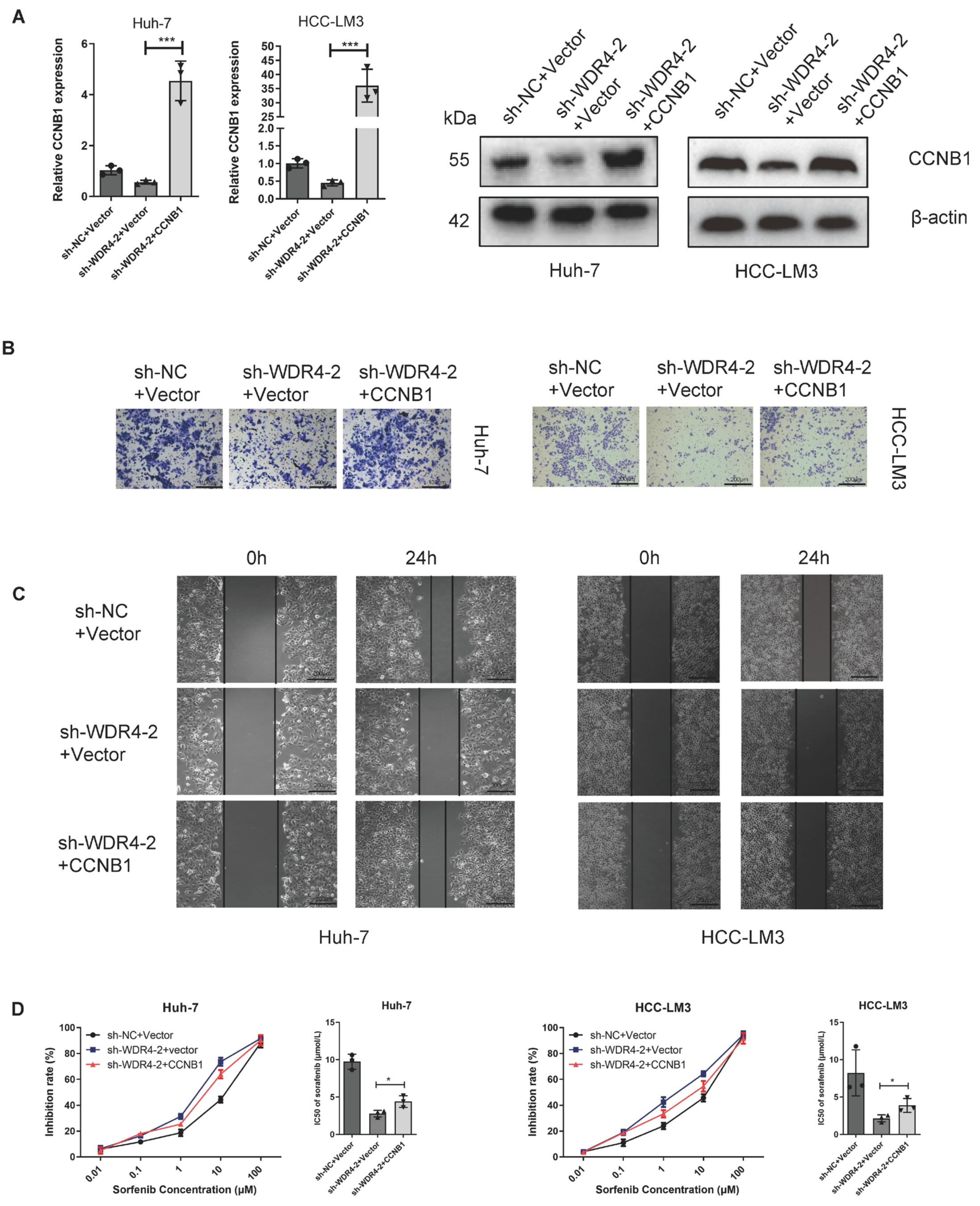

Figure S5. CCNB1 promotes HCC invasion and migration

A The CCNB1-overexpression plasmid was transformed into Huh-7 cells and HCC-LM3 cells with stable WDR4 knockdown. B Transwell Matrigel invasion assay of cell invasion ability. C Scratch wound-healing motility assay of cell migration. D The effect of CCNB1 overexpression on sorafenib resistance.

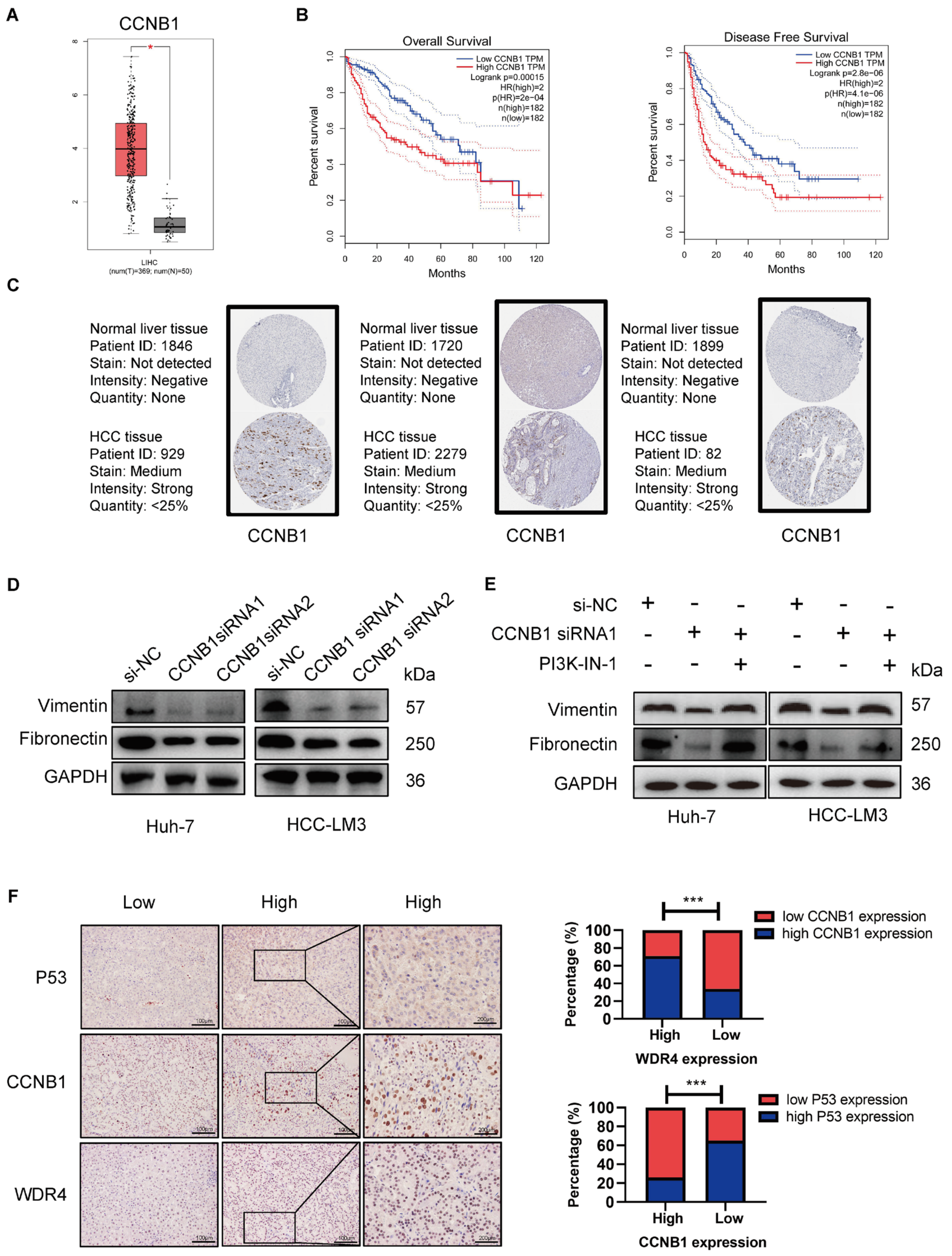

Figure S6. CCNB1 is highly expressed in HCC

A, B Analysis of CCNB1 transcript levels and the overall survival curve of HCC patients. C The HPA database shows that CCNB1 expression in HCC tissues is significantly higher than that observed in normal liver tissues. D, E Western blot results showing changes in the protein expression of EMT. F P53, CCNB1 and WDR4 expression were determined by IHC analysis.
