# Supplementary material for: MYC-targeted WDR4 promotes proliferation, metastasis, and sorafenib resistance by inducing CCNB1 translation in hepatocellular carcinoma
Source: Cell Death Dis. 2021 Jul 9;12(7):691. doi: 10.1038/s41419-021-03973-5 (PMC8270967; doi:10.1038/s41419-021-03973-5)
Supplement: Supplementary file 2 — Supplementary tables S1−S5 [file 41419_2021_3973_MOESM2_ESM.pdf]

Supplementary Table S1 Correlation between clinicopathological features and WDR4 expression in HCC tumor tissues.

Characteristics		Number	WDR4 expression		P value
			High	Low	
Gender					0.1147
	Male	45	19	26	
	Female	35	21	14	
Age					0.4912
	＜65	49	26	23	
	≥65	31	14	17	
HBV infection					0.4586
	No	23	10	13	
	Yes	57	30	27	
AFP					0.8161
	≤400 (μg/L)	29	15	14	
	＞400 (μg/L)	51	25	26	
Tumor size					***0.0256******
	≤5cm	39	15	24	
	＞5cm	41	26	15	
TNM staging					***0.0015******
	I-II	46	16	30	
	III-IV	34	24	10	
Lymph node metastasis					***0.0222******
	Absent	59	25	34	
	Present	21	15	6	
BCLC stage					***0.0170******
	Low	54	22	32	
	High	26	18	8	
PVTT					***0.0251******
	NO	38	14	24	
	YES	42	26	16	

*The expression of WDR4 were compared between the tumor tissue and the normal tissue. BCLC, Barcelona Clinic Liver Cancer; PVTT, portal vein tumor thrombus. Bold italics indicate statistically significant values. *P<0.05

Supplementary Table S2 Univariate and multivariate analyses of clinicopathological characteristics, and WDR4 with overall survival.

Characteristics	Univariate analysis			Multivariate analysis		
	Hazard Ratio	95% CI	P value	Hazard Ratio	95% CI	P value
Gender	1.662	0.983-2.809	0.058			
Age	0.749	0.437-1.284	0.294			
HBV infection	1.080	0.599-1.946	0.798			
AFP (μg/L)	0.892	0.517-1.537	0.680			
Tumor Size (cm)	1.780	1.041-3.045	***0.035****	1.490	0.839-2.646	0.174
TNM staging	2.277	1.342-3.864	***0.002****	1.114	0.534-2.322	0.774
Multiple nodules	3.482	1.884-6.435	***<0.001****	1.368	0.592-3.161	0.463
BCLC stage	3.893	2.192-6.915	***<0.001****	2.198	1.010-4.782	***0.047****
PVTT	2.735	1.535-4.873	***0.001****	1.388	0.692-2.783	0.355
WDR4	4.097	2.163-7.759	***<0.001****	2.215	1.088-4.513	***0.028****

HR, Hazard ratio; CI, Confidence interval. Bold italics indicate statistically significant values. *P<0.05

Supplementary Table S3 Online analysis website

website	URL
GEPIA	http://gepia.cancer-pku.cn/
UALCAN	http://ualcan.path.uab.edu/index.html
CCLE	https://portals.broadinstitute.org/ccle/about
cBioPortal	https://www.cbioportal.org/
SIFT	http://provean.jcvi.org/index.php
PolyPhen-2	http://genetics.bwh.harvard.edu/pph2/index.shtml
GnomAD	https://gnomad.broadinstitute.org/
HPA	https://www.proteinatlas.org/
Ubibrowser	http://ubibrowser.ncpsb.org.cn/ubibrowser/home
TCGA-LIHC	https://portal.gdc.cancer.gov/
GEO(GSE105130)	https://www.ncbi.nlm.nih.gov/geo/

Supplementary Table S4 Primer sequences, siRNAs and shRNA used in this study.

Primer name		Sequence (5’-3’)
WDR4	F	TCTCCAAGTCTGGCCGCTAT
	R	CGCACCACCATCCTGACACT
CCNB1	F	GCACTTTCCTCCTTCTCA
	R	CGATGTGGCATACTTGTT
MYC	F	GTCAAGAGGCGAACACACAAC
	R	TTGGACGGACAGGATGTATGC
E-cadherin	F	CGAGAGCTACACGTTCACGG
	R	GGGTGTCGAGGGAAAAATAGG
N-cadherin	F	AGCCAACCTTAACTGAGGAGT
	R	GGCAAGTTGATTGGAGGGATG
Vimentin	F	GACGCCATCAACACCGAGTT
	R	CTTTGTCGTTGGTTAGCTGGT
Fibronectin	F	TTATGACGACGGGAAGACCT
	R	GCTGGATGGAAAGATTACTC
GAPDH	F	GGTGAAGGTCGGAGTCAACG
	R	TGGGTGGAATCATATTGGAACA
WDR4 Primer1	F	GAGGAGTCTGCTATCAAGTAA
	R	TCACGAGGTCAGGAGATG
WDR4 Primer2	F	CTGCCAACGCTAATACCA
	R	TGCCACCTCAATGAATCG
si-NC		UUCUCCGAACGUGUCACGUTT
siWDR4-1		GGACGUGGCUUUCGAGGAG
siWDR4-2		GCAGACAGCAGUUGGUGUA
siCCNB1-1		CCATTATTGATCGGTTCAT
siCCNB1-2		CCACATCGAAGCATGCTAA
siMYC-1		GAGGUAUCUGGAAGAAAUTT
siMYC-2		GGAACAAGAAGAUGAGGAATT
si EIF2A-1		GAUGUAGUUUGGAAUUCUAGU
si EIF2A-2		GGUGCACCUUCAUUUGUUAGA
shWDR4-1		GCACCGTGTTAAAGAAAGTCT
shWDR4-2		AGAGTTTGTGAGCCGTATCTC
Sh-Ctrl		TTCTCCGAACGTGTCACGT

Supplementary Table S5 Primary antibodies used in this study

Antigens	Manufacturer	Catalog Number	Application
WDR4	Abcam	ab169526	1:1000 for WB;<br>1:500 for IHC
WDR4	Abcam	ab241297	IP
CCNB1	Proteintech	55004-1-AP	IP
CCNB1	Proteintech	67686-1-Ig	1:1000 for WB
β-actin	Proteintech	66009-1-Ig	1:1000 for WB
GAPDH	Proteintech	60004-1-Ig	1:10000 for WB
MYC	Proteintech	67447-1-Ig	1:1000 for WB
EIF2A	Proteintech	11233-1-AP	IP
EIF2A	Proteintech	66482-1-Ig	1:1000 for WB
Fibronectin	Proteintech	15613-1-AP	1:1000 for WB;<br>1:500 for IHC
Vimentin	Proteintech	10366-1-AP	1:1000 for WB;<br>1:500 for IHC
E-cadherin	Proteintech	20874-1-AP	1:1000 for WB;<br>1:500 for IHC
N-cadherin	Proteintech	22018-1-AP	1:1000 for WB;<br>1:500 for IHC
CDC25	Proteintech	16485-1-AP	1:1000 for WB
P21	Proteintech	10355-1-AP	1:1000 for WB
Caspase 3	Proteintech	19677-1-AP	1:1000 for WB
Caspase 7	Proteintech	27155-1-AP	1:1000 for WB
Caspase 9	Proteintech	10380-1-AP	1:1000 for WB
7-Methylguanosine	Biovision	6655-100	Me-RIP
IgG	Servicebio	GB23303	IP
IgG	Servicebio	GB23301	IP
HRP	IPKine	A25222	IP
